# Applied Behavior Analysis at a Crossroads: Reform, Branding, and the Future of Behavior Analysis

**DOI:** 10.1007/s40614-025-00462-4

**Published:** 2025-06-11

**Authors:** Jessica Graber, Abraham Graber

**Affiliations:** 1 Wildflower Pediatric Psychology LLC, Columbus, OH USA; 2 Ohio State University Wexner Medical Center, Columbus, OH USA

**Keywords:** Reform, Philosophic doubt, Social validity, Branding, Ethics

## Abstract

In recent years, public concerns about applied behavior analysis (ABA) have intensified. This article argues that foundational principles of ABA require behavior analysts to take seriously these concerns and actively work to improve our practices. We provide an overview of ongoing reform efforts and examine how these efforts have led to the emergence of distinct brands within the field. Although these reformist efforts signal a commitment to ethical progress, they also raise concerns about the proliferation of competing credentials, conferences, and professional affiliations, which could increase confusion among clients, practitioners, and policymakers. We argue that although branding within ABA may be an expected and ultimately benign response to shifting ethical and practical norms, it also carries the potential to faction reformist efforts and further divide the field, rather than promote our collective advancement toward ever-better practices. Thus, our goals are to: (1) raise awareness around the potentialities of siloing advancements under competing labels, and (2) suggest that an integrated approach synthesizing reformist insights into a cohesive framework may have the greatest impact on advancing the whole field’s understanding of best practice.

## Introduction

Over the past decade, we have observed a surge in public concern about applied behavior analysis (ABA). Concerns have arisen amidst the neurodiversity movement, with specific emphasis on testimonials of autistic persons describing adverse effects from their prior participation in ABA (e.g., Anderson, 2023; Graber & Graber, 2023; Leaf et al., 2022a, b). We begin this article by demonstrating that foundational principles of ABA require that behavior analysts actively work to improve our practices in response to these concerns. Such a process, involving organized actions to improve the status quo (especially relative to social problems or injustices) is commonly referred to as *reform*, a term recently applied to the process of changing ABA practice (Chapman & Bovell, 2022). We provide an overview of recent reform efforts, analyze how reform efforts are developing into specific “brands” (Keenan, 2025, p. 1), and close with broad strokes reckoning about how this changing landscape may be shaping the future of our discipline.

### Philosophic Doubt, Social Validity, and Reform

Behavior analysts’ responses to the recent surge in concerns about ABA can be understood as falling within three general categories: ignore, refute, or reform. The ignore response involves neither critically evaluating nor modifying one’s research/practice in response to concerns, nor attempting to rebut concerns. The refute response involves casting doubt on the validity of the concerns, essentially discounting the credibility of the testimony on which the concerns are based. Finally, the reform response involves critically evaluating and refining one’s practice/research to address concerns about ABA raised by members of the autistic community. In determining the most appropriate response, one can look to guiding principles of our field, namely: *philosophic doubt* and *social validity.*

*Philosophic doubt* is foundational to ABA’s theoretical and applied roots (e.g., Cooper et al., 2020; Deitz, 1982; Weiss, 2018). Philosophic doubt involves humility about what we know and openness to questioning the veracity of even our most deeply held beliefs when faced with countervailing evidence (e.g., Deitz, 1982; Weiss, 2018; Zane et al., 2023). In Skinner’s (1965) words, “science is a willingness to accept the facts even when they are opposed to wishes” (p. 12). Philosophic doubt requires following the data, even when it leads to inconvenient conclusions.

The practice of philosophic doubt must, however, be approached with care. The selective application of philosophical doubt can constitute a profound epistemic vice, shielding one’s own views from critical examination while justifying the rejection of views to the contrary. For example, just as a researcher dedicated exclusively to randomized controlled trials (RCTs) may (wrongfully) judge single case design as meaningless to public health, a behavior analyst committed to systematically collected data may be disinclined to recognize the social validity data captured within unsolicited client testimony.

The importance of *social validity* (Baer et al., 1987) to ABA is reflected in our ethics code, which requires “always placing clients’ interests first,” and treating clients with “compassion, dignity, and respect” (Behavior Analyst Certification Board [BACB], 2020, p. 6). Several prominent disability and/or autism advocacy organizations have, however, characterized ABA as doing the opposite. For example, the National Council on Independent Living (NCIL) passed a resolution describing ABA as “a harmful and abusive practice,” which should be rejected in all forms (NCIL, 2021). Autistic Collaboration (n.d.), LGBTQNation (Rubino, 2021), and ThrivingAutistic (n.d.) have similarly condemned ABA. Although substantial demand (BACB, 2025b) and support for ABA remains (e.g., Lutz, 2025), there is nonetheless reason to be concerned that the field’s social validity is deteriorating, at least for some former clients, several autism community organizations, and those who devalue ABA based on their reports.

Ignoring or discounting concerns raised by members of the primary population served by our field (BACB, 2025b) seems antithetical to our foundational principles of social validity and philosophic doubt, which call on us to be responsive to new information that challenges our assumptions, including (and especially) reports of harm perpetrated within ABA. In what follows we thus operate on the assumption that reform is the most appropriate path forward.

### Moving toward Reform, and Tacting It

Responding directly to prominent points of concern, many behavior-analytic research programs have called to: (1) remove forms of aversive control, including replacing compliance-focused procedures with assent-based practices (e.g., Abdel-Jalil et al., 2023; Mathur et al., 2024; Linnehan et al. 2023; Scallan & Rosales-Ruiz, 2023); (2) explicitly center empathy and compassion (e.g., Abdel-Jalil et al., 2023; Denegri et al., 2023; Ghaemmaghami et al., 2024; LeBlanc et al., 2020; Melton et al., 2023; Penney et al., 2023; Rodriguez et al., 2023; Rohrer et al., 2021; Rohrer & Weiss, 2023; Sadavoy & Zube, 2021; Taylor et al., 2019; Wentz et al., 2023); (3) minimize risks of emotional/psychological harms, including acknowledging and preventing trauma (e.g., Arthur et al., 2023; Leyman et al., 2025; Luiselli et al., 2024; Rajaraman et al., 2022a, 2022b; Tarbox et al., 2023; Wheeler et al., 2024); and (4) actively empower clients through neurodiversity-positive practices (e.g., Graber & Graber, 2023; Linnehan et al., 2023; Mathur et al., 2024; Veneziano & Shea, 2023).

As approaches to reform burgeon, new names have been introduced, reinforced, and solidified within the common vernacular of ABA. These include: (1) *Trauma-informed behavior analysis* (e.g., Austin et al., 2024; Beaulieu et al., 2024; Beltran, 2023; Morgan, 2022); (2), *Compassion-focused ABA* (e.g., Abdel-Jalil et al., 2023; Denegri et al., 2023; Ghaemmaghami et al., 2024; LeBlanc et al., 2020; Melton et al., 2023; Penney et al., 2023; Rodriguez et al., 2023; Rohrer et al., 2021; Sadavoy & Zube, 2021; Wentz et al., 2023); (3) *Assent-based ABA* (e.g., Breaux & Smith, 2023a, b; Mead Jasperse et al., 2023); (4) *Progressive ABA* (e.g., Ferguson & Milne, 2023; Leaf et al., 2016a, b); and (5) *Today’s ABA* (Hanley, 2021; Lambert, n.d.; PFA & SBT Community, 2025; ThinkPsych, 2023).

Paralleling the proliferation of new names within ABA, there is a growing infrastructure for supporting and disseminating aligned research. For example, there are now new annual and biennial conferences, including (1) *Assent Con*, 2nd (Sex Ed Continuing Ed., 2024); (2) *Annual Trauma Informed Practice in ABA* (Bay Path University, n.d.); (3) *Upstate Caring Partners Compassionate Care Conference* (Upstate Caring Partners, 2024); (4) *Rejected 2024* (DoBetter Collective, 2025b); and (5) *Annual Constructional Approach to Behavior Analysis Conference* (Endicott College’s Institute for Applied Behavioral Sciences, 2024).

Although much of this reformist work represents diverse researchers and clinical groups, there has also been significant influence from some ABA organizations/businesses, such as the *Do Better Collective, Progressive Behavior Analyst Autism Council (PBAAC)*, and *FTF Behavioral Consulting (FTF)*. Beyond sponsoring conferences, these have their own annual membership packages (e.g., DoBetter Collective, 2025a), credentialing processes (e.g., Progressive Behavior Analyst Autism Council [PBAAC], 2025a; FTF Behavioral Consulting [FTF], 2025a) and/or codes of ethics (e.g., PBAAC, 2025d). For example, the PBAAC offers three potentially sequential credentials, each requiring its own application, exams, and fees. It should be noted that a credential from the BACB (i.e., RBT, BCaBA, BCBA, or BCBA-D) is not a requirement for any PBAAC credential (PBAAC, 2025a, b, c).

Likewise, FTF Behavioral Consulting offers seven credentials, representing increasingly advanced training and experience with the *practical functional assessment* (PFA) and *skill-based treatment* (SBT), both developed by the organization’s leadership. Providers at each level are encouraged to “level up” as their experiences with FTF’s training and methodology advance over time (FTF, 2025c). None of FTF’s certifications require a credential from the BACB (FTF, 2025a).

### Brands of ABA: A Source of Confusion

Keenan (2025) raised the concern that “branded versions of ABA” (p. 1) can create confusion among insurers, regulators, and legislators, which raises broader questions about the benefits and downsides of factions within ABA. Consider, for example, new and distinct credentialing options. BACB credentials are well-established and hold certificants accountable to unified code(s) of ethics. The PBAAC and FTF organizations offer a combined total of 10 distinct credentials. These newer, reform-oriented credentials may “help increase consumer confidence” (FTF, 2025b), but they may also “create confusion among practitioners and patients” alike (Kaplan & Shaw, 2011, p. 296).

What should it mean to a client or an employer that a practitioner has a credential from the PBAAC or from FTF, but not from the BACB? When assessing options for care, when should clients look for the PBAAC credential, the FTF credential, or neither? What does all of this mean for a state-licensed BCBA who is only credentialed by the BACB? How should a client search for sources that can, without significant conflict of interest, provide answers to these questions?

Furthermore, there is at least some evidence that credentials may cause harm while doing little to improve quality of care. Citing McLaughlin et al. (2017), Hantula (2022) notes that “licensure and certification across occupations shows a positive effect on quality in 16% of instances, a negative effect in 21% and no effect in 63%” (p. 114). In addition, licensure and certification “are shown to increase entry barriers, stifle competition, shut willing workers out of the labor force, raise prices for consumers and increase wages for those who are credentialed” (Hantula, 2022, p. 114; see Kleiner, 2017).

Even in the absence of credentials, related problems are likely to arise. What should a client make of the fact that a BCBA emphasizes an assent-based approach, but does not reference trauma-informed ABA? How should a client understand the distinction between Progressive ABA, Today’s ABA, and compassion-focused ABA? And, as Keenan (2025) highlights, what are regulators to make of the proliferation of many similar yet distinct labels?

Such questions are not merely hypothetical. Take, for example, Naturalistic Developmental Behavioral Interventions (NDBI), an ABA-based early intervention approach whose naming distinguished it as being more “naturalistic” and developmentally informed than discrete trial teaching, the predominant form of ABA-based early intervention at the time (Jobin & Schreibman, 2025). Though NDBI is one approach to implementing ABA (Schreibman et al., 2015), substantial confusion about the relationship between NDBI and ABA remains. It is common for health professionals—including psychologists—to deride “ABA” while touting the virtues of NDBI. There is some reason to think that this confusion is comparatively common in the autistic community (e.g., Dwyer, 2018). Even within some peer-reviewed behavior-analytic literature, there is a lack of clarity around the extent to which NDBI is a part of, rather than apart from, ABA (e.g., Pickard et al., 2024). If even academic behavior-analytic literature lacks clarity about the extent to which NDBI is an extension of ABA, what hope do clients and regulators have of understanding and assessing the differences between assent-based ABA, trauma-informed ABA, compassion-focused ABA, Progressive ABA, Today’s ABA, and ABA services that make no claim to being any of these?

### Brands of ABA: A Potential Challenge for Behavior-Analytic Progress

Consider the widely read work of Tarbox et al. (2023), in which common approaches to extinction are reformed to center compassion and connection, while decreasing the risks of side effects, and without sacrificing effectiveness. Though Tarbox et al. report preliminary findings, if evidence (including ethical considerations) continue to support this reformed approach to extinction, it does not follow that we should tact their protocol “kind extinction.” Instead, we should update our understanding of best practice: i.e., there is extinction done better (with kindness), and extinction done worse.

Likewise, if the evidence (including ethical considerations) supports the prioritization of assent in ABA, it does not follow that there would be any such thing as “assent-based ABA.” There would only be ABA done better (with assent prioritized), and ABA done worse. If the evidence, including ethical considerations, supports trauma-informed behavior analysis, it does not follow that there would be any such thing as “trauma-informed ABA;” only ABA done better (trauma-informed), and ABA done worse.

Brands exist to help consumers discriminate between choices (Bastos & Levy, 2012). Branding currently largely serves as a signal of reforming behavior-analytic approaches to advance our understanding and/or dissemination of ever-better practices. This critical work should not, however, happen in silos. The various methods for improving ABA documented in the academic literature are likely key, and related, ways to improve behavior-analytic research and practice. Behavior analysts and their clients are thus best served by collaborative efforts to advance our field, thereby benefiting the greatest number of people.

### One Function of Branding: Value Alignment

Consumers are drawn to brands that align with their values (Liu & Getz, 2024). Increasingly vocal criticism of “ABA” as a brand itself may thus lead some behavior analysts to feel compelled to create or align with a brand that better centers their values. Association for Behavior Analysis International’s (ABAI) recent decision to condemn the use of Contingent Electric Skin Shock (CESS), and the process by which that decision was made (ABAI, 2022c), offers a case study.

A range of consumers, behavior analysts, and affiliate organizations demanded that ABAI formally condemn CESS, and thereby distance itself (and thus “ABA”) from it (see, e.g., Association of Professional Behavior Analysts [APBA], 2022; International Association for the Scientific Study of Intellectual & Developmental Disabilities [IASSIDD], 2018; Massachusetts Association for Applied Behavior Analysis [MassABA], 2021; Oregon Association for Behavior Analysis [ORABA], 2021). ABAI initially proposed a narrow endorsement of CESS (ABAI, 2022b), followed shortly thereafter by “Final Recommendations” which continued to support the use of CESS under some narrow circumstances (ABAI, 2022a). ABAI’s initial resistance to a universal condemnation of CESS led “some members and chapters [to] quit the association” (Perone, 2023, pp. 1–2).

This saga illustrates the potential harm of value misalignment. Where there is value discordance between behavior analysts and “ABA” qua brand, behavior analysts may seek to align themselves with a brand that more explicitly corresponds with their values. This is a point that has, perhaps, not received its due.

Consider, for example, Perone’s (2023) argument that ambiguity remains regarding the social validity of CESS. Perone appears, however, to overlook one important set of stakeholders: behavior analysts (or, in particular, “a significant majority of ABAI members who cast votes” (Lerman, 2023, p. 314)). Here, one can confidently answer “yes” to the question, “... is it really obvious that CESS lacks social validity?” (Perone, 2023, p. 309). The vote was taken; the tally is in. Whether there will be lasting effects to the debate surrounding CESS remains to be seen. Will those individual and groups that left ABAI during the debate return? Will there be enough new members to balance the numbers lost? What effect, if any, will all of this have on continued proliferation of new “brands” of ABA?

The BACB’s decisions to “no longer include the integration of DEI into any of the course-content areas” and to remove the CEU requirement on DEI (BACB, 2025a, p. 2) threatens a similar schism. This choice has been met with fierce opposition (Black Applied Behavior Analysts [BABA], 2025; Schwartz, 2025). In the short-term, the BACB’s decision will reshape the way behavior analysts are trained. This will likely be true even for programs that wish to maintain an emphasis on DEI. For example, recent legislation in Ohio bans DEI from higher education *except* where DEI programs are required for accreditation (136 th General Assembly, 2025). In Ohio and states with similar laws, the BACB’s decision to remove DEI from coursework requirements effectively prevents behavior analytic programs from incorporating DEI into their curriculum.

In addition to the potentially detrimental impact that the BACB’s decision will have on marginalized populations (BABA, 2025), the decision may also lead to further brand proliferation. For example, the Black Applied Behavior Analysts (BABA) Response to the Behavior Analyst Certification Board (BACB) Removal of DEI Requirements^1^ calls for behavior analysts to “Leverage Alternative Certification and Accreditation Bodies” to combat the erasure of DEI (BABA, 2025, para. 23). The BACB’s choice regarding DEI coursework requirements must, first and foremost, be evaluated based on its impact on marginalized communities; the impact on “ABA” as a brand is only a secondary consideration. Nonetheless, it seems likely that the BACB’s decision will further contribute to brand proliferation in ABA.

## Conclusions

The growth of new behavior-analytic brands may be both inevitable and benign. The proliferation of brands may be inevitable because new labels are needed to differentiate emerging trends in ABA from their predecessors. The proliferation of brands may also be benign because it may be a temporary step along the path to an emerging consensus regarding the roles of assent, compassion, cultural responsiveness, and trauma-informed care in best practice.

Nonetheless, confusion regarding the relationship between NDBI and ABA presages a future in which, rather than emerging behavior-analytic brands ultimately remerging to contribute to a new understanding of best practice, they continue to grow apart. Clients and providers are forced, without easy access to unbiased information, to choose among brands with competing costs and/or credentialing systems, and the field becomes increasingly siloed. In this scenario, rather than coming to a unified understanding, we are left with disparate and competing brands, each characterized by a myopic emphasis on only part of the richly textured tapestry that constitutes behavior-analytic best practice.

Awareness, intentionality, and authenticity are recommended to chart a course toward a positive future for the field. This involves taking seriously the ebbing social validity of ABA and considering the extent to which our own behaviors (as providers, researchers, and/or organizational leaders) may be contributing to this outcome. For those doing reformist work, rather than conceptualizing this work as a new and distinct kind of ABA, we can view this work as continuous with, while nonetheless improving upon, traditional practice. Journal editors and reviewers can also help steer the rising tide of branding by encouraging cross pollination between different approaches to ABA engaging in the project of reform. So long as diverse, progress-oriented approaches to ABA constructively engage with one another within the peer-reviewed literature and at professional conferences, we are unlikely to encounter the worst potentialities of branding. In the end, when it comes to how we treat our clients, there is better ABA, and there is worse ABA. Let’s all strive, together, to move the field in the direction of the better.

## Data Availability

There are no data associated with this manuscript.

## Footnotes

1 Both authors signed on to this document.
